# HPV16 Entry into Epithelial Cells: Running a Gauntlet

**DOI:** 10.3390/v13122460

**Published:** 2021-12-08

**Authors:** Snježana Mikuličić, Johannes Strunk, Luise Florin

**Affiliations:** Institute for Virology and Research Centre for Immunotherapy (FZI), University Medical Centre of the Johannes Gutenberg-University Mainz, Obere Zahlbacher Strasse 67, 55131 Mainz, Germany; snmikuli@uni-mainz.de (S.M.); johannes.strunk@uni-mainz.de (J.S.)

**Keywords:** human papillomavirus, virus entry, HPV, HPV16, L1, L2, endocytosis, trafficking, intrinsic immunity, restriction factor

## Abstract

During initial infection, human papillomaviruses (HPV) take an unusual trafficking pathway through their host cell. It begins with a long period on the cell surface, during which the capsid is primed and a virus entry platform is formed. A specific type of clathrin-independent endocytosis and subsequent retrograde trafficking to the trans-Golgi network follow this. Cellular reorganization processes, which take place during mitosis, enable further virus transport and the establishment of infection while evading intrinsic cellular immune defenses. First, the fragmentation of the Golgi allows the release of membrane-encased virions, which are partially protected from cytoplasmic restriction factors. Second, the nuclear envelope breakdown opens the gate for these virus–vesicles to the cell nucleus. Third, the dis- and re-assembly of the PML nuclear bodies leads to the formation of modified virus-associated PML subnuclear structures, enabling viral transcription and replication. While remnants of the major capsid protein L1 and the viral DNA remain in a transport vesicle, the viral capsid protein L2 plays a crucial role during virus entry, as it adopts a membrane-spanning conformation for interaction with various cellular proteins to establish a successful infection. In this review, we follow the oncogenic HPV type 16 during its long journey into the nucleus, and contrast pro- and antiviral processes.

## 1. Introduction

Human papillomaviruses are causally associated with multiple human cancers, including cervical as well as head and neck cancer. They are small DNA tumor viruses that infect the epithelium of skin and mucosa. Based on their potency to induce cancer, which was initially described by Harald zur Hausen [1], HPVs are divided into low- and high-risk HPV types. Low-risk types cause benign lesions, while high-risk types, also termed as oncogenic or carcinogenic HPVs, can immortalize epithelial cells. Therefore, high-risk types are declared as causative agents for premalignant and malignant changes [2,3]. HPVs are highly prevalent in the human population, but most infections are under tight control of the immune system and therefore asymptomatic and self-resolving. On the other hand, HPV has evolved mechanisms to evade host immune defenses enabling persistent infections, virus replication, and oncogenesis [3,4,5,6,7].

Structurally, papillomaviruses are nonenveloped viruses of about 55 nm in diameter. The icosahedral capsid has been shown to have an enormous degree of flexibility and is composed of 360 copies of the major capsid protein L1 and 12–72 copies of the minor capsid protein L2 and contains the approximately 8 kb circular double-stranded DNA genome [8,9,10,11,12].

The HPV replication cycle involves three successive major steps: (1) virus entry including delivery of the viral genome to the host-cell nucleus, (2) viral gene expression and genome replication, (3) and eventually virus assembly and release. Expression of the viral genes, especially of the late genes including L1 and L2, is linked to differentiation processes of the epithelium. For this reason, it is difficult to propagate papillomaviruses in cell culture. To circumvent this limitation, the majority of papillomavirus research is based on the substitutes of naïve viral particles, such as virus-like particles (VLPs), pseudovirions (PsVs), and quasivirions (QVs) [13]. VLPs are composed of the viral capsid proteins L1 and L2, whereas PsVs and QVs, in addition to the capsid, contain a pseudogenome encoding a reporter gene or a modified HPV genome, respectively. Recently generated LCR-PsVs, in which the long control region (LCR) of the HPV genome regulates the reporter gene, allow quick and biologically safe particle production to track infection including the role of transcription factors during early viral gene expression [14].

**Figure 1:**
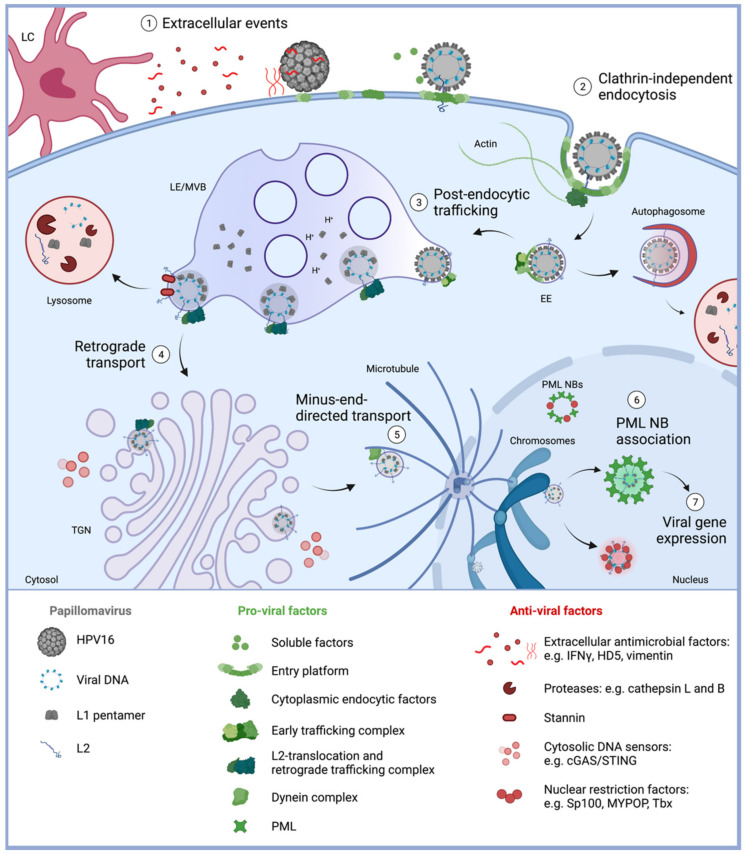
Diagram of HPV16 entry into their host cells with a focus on host restriction factors. (**1**) Extracellular events. HPV16 requires mitotically active epithelial cells for a successful infection and subsequent replication. However, various defense mechanisms form a physical, chemical or biological barrier to prevent viral access. This includes nondividing cells, extracellular antimicrobial factors, such as HD5 and vimentin, and innate immune cells including Langerhans cells (LC). Nevertheless, virions gain access through epithelial wounding, allowing the attachment to virus receptors, conformational changes of the capsid, and formation of an entry platform. (**2**) Clathrin-independent endocytosis. HPV enter epithelial cells via an unconventional clathrin-independent endocytic pathway that involves multiple signaling events, resulting in transmembrane protein clustering, recruitment of cytoplasmic endocytosis factors, and induction of actin dynamics. These events induce inward budding and fission of the membrane to noncoated vesicles. (**3**) Post-endocytic trafficking. Once inside the cell, trafficking adaptors form an early trafficking complex to facilitate early endosome (EE) maturation and virus trafficking to late endosome/multivesicular bodies (LE/MVB). The low pH inside of LE/MVBs facilitates capsid disassembly. Stable L2 insertion into the endosomal membrane is assisted by a translocation complex and allows further trafficking and virus escape from autophagosomal and lysosomal degradation by proteases. (**4**) Retrograde transport. L2 interaction with protein complexes involved in retrograde trafficking fulfill the subsequent virus transport to the TGN. With L2 as the only viral component being exposed to the cytosol, the infectious complex evades cytosolic DNA sensors, including the cGAS/STING pathway. The antiviral transmembrane protein stannin can induce sorting of HPV16 to lysosomes and virus degradation. (**5**) Minus-end directed transport. With the onset of mitosis, the fragmentation of the TGN and breakdown of the nuclear membrane allow the release and transport of membrane-encased virions towards mitotic chromatin. Here, the minus-end directed transport along microtubules is dependent on L2 interaction with the dynein motor complex and components of nuclear import machinery. Tethering of the virus vesicle to mitotic chromatin facilitates the localization inside the nucleus after completion of mitosis. (**6**) PML NB association. Inside the nucleus, released viral DNA can be detected and repressed by nuclear restriction factors, such as Sp100 and MYPOP. HPV16 association with reassembling PML NBs after mitosis leads to modified virus-associated PML NBs, probably providing a protective environment for the establishment of infection and (**7**) viral gene expression. Created with BioRender.com (accessed on: 12 November 2021).

In this review, we focus on the first step of the HPV16 replication cycle, including the complex extracellular and intracellular processes of HPV16 entry into epithelial cells and the viral strategies to overcome various cellular antiviral defense mechanisms (see Figure 1).

## 2. Extracellular Events

The extracellular events comprise virus translocation across physical barriers, attachment to mitotically active cells, conformational changes of the capsid, and formation of the entry platform.

The mucous epithelium represents the first barrier. Secretion of viscous fluid containing enzymes and antimicrobial peptides (extracellular antimicrobial factors), along with layers of nondividing cells, can impede access to basal mitotically active cells of the epidermis. Wounding facilitates the access and exposes virus-attachment factors on the basement membrane or on the cell surface of basal cells to initiate infection [15]. Interactions of positively charged lysine residues of the major capsid protein L1 with negatively charged heparan sulfate proteoglycan (HSPG) cause conformational changes of both capsid proteins, which in turn may weaken the virus bond to these glycans [16,17,18,19,20,21]. Further conformational changes of the capsid are mediated by the peptidyl-prolyl cis/trans isomerase cyclophilin B (CyPB) and lead to exposure of the L2 N-terminus [22]. These initial changes in the virus structure not only enable the capsid to bind to downstream entry factors such as priming proteases but also might provide a window for antimicrobial peptides such as α-defensins to attack (cellular defense mechanisms and proteins are summarized in Table 1).

Additional modifications in the capsid structure induced by proteolytic cleavage of HPV16 L1 and L2 by the secreted trypsin-like serine protease kallikrein-8 (KLK8) and the proprotein convertase furin, respectively, have been suggested to contribute to the slow and asynchronous internalization kinetics and to promote subsequent steps of the infectious entry pathway [23,24,25,26,27,28].

Parallel to structural changes on the capsid, HPV-induced together with virus-independent signaling events via HSPGs, integrins, and growth factor receptors (GFRs) support the HPV16 entry pathway [29,30,31,32,33,34,35]. Host-cell sheddases such as ADAM17, a disintegrin and metalloproteinase 17, cleave the proform of various membrane-bound signaling molecules that bind to GFRs and activate the mitogen-activated protein kinase (MAPK) ERK1/2 signaling pathway. The efficiency of ADAM17-mediated ERK activation and the subsequent virus entry platform formation can be regulated by a member of the tetraspanin family, the Cluster of Differentiation 9 (CD9 or tetraspanin-29, Tspan-29), [36,37,38]. This transmembrane protein provides a scaffold for ADAM17 and its epidermal GFR (EGFR)-activating substrates [39]. Downstream of proteinases, the released growth factors (GFs) activate EGFR, which is suggested to result in the translocation of the phospholipid-binding protein annexin A2/S100A10 heterotetramer (A2t) to the outer leaflet of the plasma membrane. This process probably supports A2t interaction with L2 [40,41] and the recruitment of the preassembled entry receptor components, EGFR and tetraspanin CD151 (Tspan-24), to virus-binding sites [34]. Additional roles of cell-surface A2t, that may affect HPV infection, comprise the HPV-mediated suppression of the cell-mediated immune response by decreasing Langerhans cell maturation and the regulation of ADAM-mediated ectodomain shedding of GFs [42,43,44].

The association of GFs and cleaved HSPGs with the viral capsid may support the formation of the HPV16 entry platform. This scenario is likely, as virions are found in complexes with GFs, their receptors, and soluble HSPGs [32]. Laminin-332 and laminin-binding integrins have been shown to bind HPV16 particles and are therefore suggested as additional components of the entry receptor complex [45,46,47,48,49,50]. The tetraspanin CD151 not only associates with EGFR but also regulates integrin activities by influencing their positioning within tetraspanin-enriched microdomains [34,50,51,52,53,54,55] and is crucial for the process of HPV16 endocytosis into their host cells [56,57,58,59]. In general, diverse tetraspanins orchestrate the spatiotemporal coordination of viral entry receptor components by anchoring specific proteins to one site on the cell membrane. This results in the formation of microclusters that further organize into larger assemblies. These microdomains enable efficient cleavage of cellular and viral proteins, signaling, and membrane dynamics, leading to virus endocytosis [60,61].

In contrast to proviral factors, recognition of HPV by the innate immune system can inhibit virus entry and infection. For example, human α-defensins are a family of secreted host-defense peptides and effector components of innate immunity. Particularly, α-defensin 5 (HD5) displays its potent activity against HPV16 infection [62]. A model proposes that interaction of HD5 with the C-terminal tail of the major capsid protein L1 stabilizes the HPV16 capsid and prevents the access of the priming protease furin to its substrate, the minor capsid protein L2. Thereby, HD5 inhibits proteolytic processing of the capsid, redirects the incoming viral particles to the lysosomes, accelerates the degradation of internalized capsid proteins and restricts infection [63,64,65] (see Table 1). Vimentin and the antiviral cytokine interferon gamma (IFN-γ) might also act as virus-restriction factors. Binding of extracellular soluble vimentin to incoming HPV16 particles leads to reduced virus internalization [66]. In general, IFN-γ, is released from stimulated immune cells to induce expression of antiviral genes. However, in HPV16 infection, IFN-γ seems to act in a more direct manner. Treatment of cells with IFN-γ stimulates the JAK2/STAT1 pathway, which leads to decreased L1 proteolytic cleavage and retention of L2 and the viral genome in LE/MVBs, most likely resulting in the degradation of internalized capsid [67].

**Table 1:** Cellular defense mechanisms and antiviral factors providing intrinsic immunity against HPV16 during the entry pathway are listed in alphabetical order.

Cellular Defense	Impact on HPV16 Entry	References
Alpha-defensins	Antimicrobial peptide α-defensin 5 (HD5) binds to the C-terminal tail of L1 and negatively charged regions of L2. L1/L2/HD5 interaction inhibits L2 cleavage by furin, stabilizes the capsid, alters virus trafficking and accelerates capsid protein degradation.	[62,63,64,65]
Autophagy	HPV-mediated activation of the mTOR pathway suppresses autophagy. Inhibition of autophagy delays degradation of L1 enabling efficient infection. Reduction in L2 polyubiquitination by TSG101/L2 interaction might contribute to bypass autophagy.	[33,68,69]
Cathepsins	Internalized viruses can be routed to lysosomes and degraded by acid-dependent proteases cathepsin L or B.	[23,70]
cGAS/STING	Localization of viral DNA to the cytosol activates the cGAS/STING pathway and the activation of defense mechanisms. HPV16 DNA remains in a transport vesicle that prevents the DNA sensing.	[71]
Interferon-gamma	IFN-γ decreases L1 proteolytic priming and retains L2 in endosomes.	[67]
Langerhans cells (LC)	LC are the tissue-resident macrophages of the epithelium presenting virus antigens. HPV16 suppresses LC maturation and the cell-mediated immune response through interaction with A2t.	[44]
MYPOP	MYPOP senses incoming viruses via interaction with L2 and blocks viral early gene expression. L2 interaction with MYPOP enhances MYPOP binding to the viral DNA (LCR).	[14]
Sp100	Sp100 represses viral gene expression. HPV16 delays the recruitment of this restriction factor probably to enable initial gene expression.	[72,73]
Stannin	Stannin interacts with L1 and impairs the L2/retromer interaction and accelerates virus degradation.	[74]
Tbx2 Tbx3	T-box proteins, Tbx2 and Tbx3, repress the activity of the HPV16 LCR and might play a role in the regulation of HPV gene expression.	[75]
Vimentin	Soluble extracellular vimentin inhibits HPV16 uptake by direct binding to the incoming virus.	[66]

## 3. Clathrin-Independent Endocytosis

HPV16 particles trigger their uptake into cells by clustering components of the entry receptor complex into large entry platforms, so-called endocytic pits. As mentioned above, multiple proteins have been suggested as such components, including tetraspanins, laminin-binding integrins, growth-factor receptors, and the annexin A2 heterotetramer. Simultaneous binding of capsid proteins with entry receptor components might induce initial platform formation and membrane curvature, both required for efficient virus endocytosis.

As demonstrated by several electron microscopical analyses, inward budding of the HPV16-associated endocytic pit results in the formation of noncoated vesicles of about 100 nm in diameter [31,58,76,77]. This process is independent of the classical key factors clathrin, caveolin, and dynamin [31,57,58]. Therefore, it is believed that HPV16 initiates or follows a novel endocytic pathway that requires tetraspanins [58] and the actin cytoskeleton [31,57,78], and shares characteristics of macropinocytosis [31].

Like entry platform formation, HPV16 endocytosis is promoted by host-cell signaling via EGFR [31,32,34,36,50,76]. Downstream of EGFR, Abelson tyrosine-protein kinase (Abl) regulates actin remodeling to inhibit macropinocytosis of EGFR, favoring endocytosis into small endocytic vesicles [79]. As such, Abelson tyrosine-protein kinase 2 facilitates HPV endocytosis [76] and supports the notion that HPV16 is cointernalized with EGFR in an actin-dependent manner.

Additionally, filamentous actin, the cytoskeletal adaptor protein Obscurin-Like 1 (OBSL1), the trafficking protein particle complex subunit 8 (TRAPPC8), the Wiskott–Aldrich syndrome protein and suppressor of cAMP receptor homologue (WASH), and the BAR domain-containing sorting nexin 2 (SNX2), have been shown to localize at the HPV16 endocytic pits [80,81,82,83]. As demonstrated for CD151, the cytoplasmic endocytic factors WASH and SNX2 are cointernalized with virus particles and may trigger actin dynamics, membrane curvature, virus internalization and vesicle scission [56,82]. This may function by complex formation with CD151 and/or OBSL1, as basolateral membrane-bound virus particles were found to be surrounded by tetraspanins, OBSL1, and actin. These findings support the idea that OBSL1 is a linker between virus-induced endocytic pits and the actin cytoskeleton [80,81]. On the other hand, the interaction of OBSL1 with L2 points towards a later function, because L2 penetrates into the cytosol most likely just before trafficking from the late endosome towards the nucleus. Alternatively, initial L2 membrane insertion might occur already on the plasma membrane, enabling L2 interaction with cytoplasmic factors required for HPV16 endocytosis and subsequent trafficking.

## 4. Post-Endocytic Trafficking to the Disassembly Compartment

After endocytosis, the HPV16 entry platform further determines the post-endocytic trafficking of the virus. For example, tetraspanins and A2t both colocalize with HPV on the cell surface and are cointernalized with the virus during endocytosis [56,58,84]. This internalization process results in the recruitment of trafficking adaptors, such as syntenin-1 and Rab5, to virus-filled endosomes [77]. Interaction of syntenin-1 with the C-terminus of CD63 (Tspan-30) promotes maturation and trafficking of the virus-filled early endosomes to late endosomes/multivesicular bodies (LE/MVB) [77]. ESCRT components such as the vacuolar protein sorting-associated protein 4 (VPS4), the tumor susceptibility 101 (Tsg101) and the ALG-2-interacting protein X (Alix) together with sorting nexin 17 (SNX17) and the cytosolic-containing TCP1 (CCT) chaperonin complex, are additional trafficking proteins that can be recruited to incoming HPV and have been proposed to play a role during this intracellular trafficking/maturation process [69,77,85,86,87,88]. Many of them have the ability to interact with L2 (see Table 2). Moreover, the sorting adaptor syntenin-1 can bind to Rab-GTPases, Rab5 and Rab7, principal regulators of endocytic trafficking [89] suggested to control endocytic routes of internalized HPV (reviewed in [90]).

Similar to tetraspanins, Rab-GTPases, and the ESCRT machinery, A2t promotes membrane dynamics and vesicular trafficking [91]. Therefore, A2t might cooperate with other trafficking factors in HPV16 entry. It was shown to associate with incoming viruses and CD63 and to promote virus trafficking to LE/MVBs [40,84]. The continuously decreasing pH inside maturing endosomes facilitates capsid disassembly, partial dissociation of L1 from the infectious complex, as well as L2 translocation across the endosomal membrane [92,93,94,95].

Alternatively, HPV can be eliminated by degradation. Autophagy-mediated clearance of intracellular pathogens (also called xenophagy) is one of the fundamental degradation pathways of the cell that contributes to antimicrobial defense [96]. The recruitment of the microtubule-associated protein 1 light chain 3 (LC3) and autophagy-related (ATG) proteins to autophagosomal membranes and polyubiquitinated targets leads to inclusion of pathogens in double-membrane vesicles called autophagosomes. Like many pathogens, HPV16 has been detected in autophagosomes [97]. On the one hand, HPV16 induces the formation of LC3 puncta and LC3 association with ATG9, suggesting induction of autophagy [68,98]. On the other hand, HPV16 has evolved ways to evade this degradation compartment. HPV-mediated activation of the phosphatidylinositol 3-kinase (PI3K) and mammalian target of rapamycin (mTOR) pathway downstream of EGFR efficiently suppresses autophagy and enables infection [33]. The TSG101-mediated reduction in L2 polyubiquitination might additionally contribute to bypassing autophagy [69].

Antiviral factors which prevent capsid priming, disassembly or L2 translocation, enhance the degradation of the capsid proteins (see Table 1) via the lysosomal pathway or autophagy. The latter also leads to proteolytic degradation of the autophagosomal content, as autophagosomes fuse with lysosomes during maturation to autophagolysosomes. Acid-dependent proteases such as cathepsins have been assumed to contribute to the antiviral degradation process [70].

## 5. L2 Translocation and Retrograde Transport

Numerous studies uncovered cellular interaction partners of the HPV16 minor capsid protein L2 (summarized in Table 2). In most cases, these L2 partners assist in L2 translocation across the host-cell membrane and subsequent retrograde transport (reviewed in [99]). As described above, the process might start early in infection, with initial conformational changes of the capsid structure allowing L2 exposure, priming and accessibility of L2 to cellular proteins. Cyclophilin B contributes not only to initial conformational changes in the capsid, but also assists in dissociation of L1 and L2 in LE/MVBs [94]. The membrane-destabilizing peptide near the C terminus of L2, which contains a cell-penetrating peptide (CPP), might initiate membrane penetration [100,101] which eventually leads to incorporation of the L2 transmembrane domain into the vesicular lipid bilayer, a luminal N-terminus and the majority of the protein being exposed to the cytosol [95,102].

The relevance of the transmembrane protease γ-secretase for the HPV entry process is well-documented [99,103,104,105,106,107,108]. Here, a novel chaperone-like function of the γ-secretase seems to be more important than its role as a protease, as it enables low pH-dependent membrane insertion of the furin-cleaved L2 that is required for further trafficking [25,104,105,106]. Additional chaperones from the Hsc/Hsp70 family can form physical complexes not only with γ-secretase but also with L2, and might therefore assist in the process of membrane insertion [104,109]. Moreover, γ-secretase is located in and regulated by tetraspanin microdomains [110]. Therefore, it is conceivable that tetraspanins provide a scaffold not only for the endocytic machinery but also for the L2-translocation complex. Consistent with this model, the cytosolic γ-secretase adaptor p120 catenin forms a complex with HPV, probably mediated via a transmembrane protein at early time points during viral internalization as well as trafficking, and promotes interaction of L2 with γ-secretase to support membrane insertion [103]. Despite many convincing studies, the exact mechanism and the role of the involved factors that mediate L2 translocation are not yet fully understood.

Recent findings suggested that the viral genome and remnants of the capsid remain in a transport vesicle during the complete intracellular transport, to evade innate immune detection by cellular restriction factors [71,111,112,113]. Only L2 extends the vesicle to hijack the cellular transport machinery [95]. Interactions of L2 with components of the retromer complex and probably a newly identified retriever complex fulfill endosome tubulation, vesicle formation, and the retrograde transport of HPV16 from the endosome to the trans-Golgi network (TGN) [92,114,115]. Rab-GTPases and numerous molecules including SNX proteins, the TBC1 domain family member 5 (TBC1D5), and the endoplasmic reticulum (ER)-anchored protein vesicle-associated membrane protein (VAMP)-associated protein (VAP), may assist in these processes [90,99,115,116,117,118]. For example, TBC1D5 is recruited to L2/retromer at the endosomal membrane, which subsequently stimulates hydrolysis of Rab7-GTP to drive retromer disassembly from the HPV [117]. After retromer dissociation, virus-containing vesicles traffic to the TGN, where the cargo is delivered by membrane fusion.

Results of one study has indicated that during the intracellular HPV16 trafficking process, the small transmembrane protein stannin is able to sense incoming viruses via interaction with L1. This interaction impairs binding of L2 to the retromer, likely by preventing L2 translocation across vesicular membranes, which again leads to routing the virus for lysosomal degradation and restriction of HPV infection [74].

## 6. Minus-End-Directed Transport to PML Nuclear Bodies

The onset of mitosis is accompanied by three cellular reorganization processes required for HPV16 transport into the host-cell nucleus and the establishment of infection [119]. The reliance of HPV16 on these processes explains the papillomavirus dependency on a cell division cycle for a successful infection.

First, the fragmentation of the TGN during mitosis promotes the release of membrane-encased virions, enabling subsequent trafficking of the virus and evasion of cellular immune defenses [71,111,120]. In contrast to viruses which release their genome into the cytoplasm, HPV16 resides in the Golgi-derived vesicle until mitosis is completed [111]. This enables the virus to escape from cytosolic DNA sensors such as the cyclic GMP–AMP synthase–stimulator of interferon genes (cGAS/STING) pathway and downstream defense mechanisms [71]. The nonreceptor tyrosine kinase Pyk2 as well as interaction of L2 with TRAPPC8 have been suggested to assist in Golgi exit and destabilization, respectively [83,121]. OBSL1, which can bind to L2′s cytoplasmic part, may contribute to this process, as OBSL1 has been found to be a critical regulator of Golgi morphogenesis [80,122]. Further work is needed to understand how HPV16 exits the Golgi or whether HPV contributes to its fragmentation.

After dissociation from the TGN, the virus–vesicle associates with microtubules via L2 interaction with the dynein motor complex [111,123,124]. This most likely facilitates the transport along astral microtubules located between the TGN and the microtubule-organizing center (MTOC). L2 interaction with dynein light chains Tctex-Type 1 and 3 (DYNLT1/Tctex1 and DYNLT3) is mediated via a consensus Tctex-interaction domain located in the C-terminus of L2 [123,124].

The second mitosis-associated reorganization process required for HPV infection is the nuclear envelope breakdown [125,126]. It enables a direct transport to the cellular DNA and opens the gate for the virus–vesicles to the cell nucleus. Here, L2 forms a physical complex not only with the dynein light chains but also with the Ran-binding protein 10 (RanBP10) and karyopherin alpha 2 (KPNA2) to promote the minus-end-directed transport of the virus towards mitotic chromatin [127]. Conserved nuclear import signals (NLS) within L2 and interactions of L2 with components of the nuclear import machinery (including KPNA2) have been shown to play a role for L2 nuclear import [128,129,130] and seem to be also required during virus entry, although the nuclear envelope is absent during mitosis. The observation that L2/Hsc70 interaction promotes the release of newly synthesized L2 from dynein complexes suggests a role of the Hsc70 chaperone for the release of incoming virus from the transport complex [123].

In contrast to earlier assumptions, parts of the L1-formed capsid appear to remain associated with L2 and the viral DNA during nuclear entry [112,113]. As L1 and the viral DNA are still hidden in the vesicle, L2 tethering to mitotic chromatin promotes the inclusion of HPV16 into the newly formed nucleus [111,120,126]. For binding to mitotic chromosomes, the virus requires a central chromosome-binding region (CBR) of the L2 protein and likely an unknown prometaphase activated factor [126]. The CBR contains a highly conserved SUMO-interacting motif (SIM) [131] that is indispensable for chromosome tethering and incorporation of the viral genome into newly formed promyelocytic leukemia (PML) nuclear bodies (PML NBs) after mitosis [73,131]. Therefore, L2 interaction with a tethering factor that is SUMO-modified during prometaphase is likely. L2 itself can also be covalently modified at its SUMO conjugation motif (SCM), and has the ability to reorganize PML NBs [130,131,132,133]. The post-translational modification is facilitated by L2 interaction with SUMO, and may exert additional functions during a so-far-undefined step in the HPV replication cycle [131,132].

Dis- and reassembly of the PML NBs is the third mitotic reorganization process required for HPV16 infection. It leads to the formation of modified virus-associated PML NBs and enables viral transcription and replication [73,134,135]. After the release of the genome in the host-cell nucleus, repressive transcription factors (restriction factors) have access to the viral DNA, or more specifically, to the HPV long control region on the HPV16 genome. The Speckled protein 100 (Sp100), transcription factors of the T-box family, Tbx2 and Tbx3, as well as the Myb-related transcription factor, partner of profilin (MYPOP) efficiently repress viral gene expression [14,72,75]. In the case of Sp100, it has been demonstrated that HPV16 delays the recruitment of this restriction factor to the viral genome and PML protein, probably to enable initial gene expression [73]. The newly identified host restriction factor MYPOP not only binds to the viral DNA but may also sense incoming viruses via interaction with L2 [14]. MYPOP interacts directly with the C-terminal part of L2 and has been observed to colocalize with L2 and the viral DNA in the nucleus after virus entry. Moreover, L2 enhances MYPOP’s binding to the viral DNA [14]. The inclusion of incoming HPV into PML NBs might play a role in protecting the viral genome from MYPOP. On the other hand, a high expression level of nuclear MYPOP in normal epithelial cells of skin and mucosa might contribute to the strong immune control of oncogenic HPV types.

## 7. Concluding Remarks

The discovery of the unconventional clathrin-independent endocytosis pathway as well as the intracellular transport of HPV16 inside a vesicle into the cell nucleus shows that viral particles are still a unique tool to uncover new endocytosis or transport routes that are most likely not only hijacked by one virus type. The formation of tetraspanin-enriched entry platforms, for example, has also been described for coronaviruses, influenza A virus or hepatitis C virus [60,136]. A protected virus transport in a Golgi-derived vesicle into the cell nucleus could also represent a more common mechanism for invading viruses to evade cytoplasmic sensors for foreign nucleic acids. Studies on viral proteins have long contributed to the discovery and characterization of cellular proteins and pathways. The most famous example of HPV is certainly the discovery of p53 by investigating the HPV E6 protein [137,138]. Interaction screens for L2 not only contributed significantly to the elucidation of the HPV16 entry pathway but also led to the identification of the previously unknown viral restriction factor MYPOP. Future studies must show whether this transcription factor has more general functions in cellular defense, virus latency or even in preventing oncogenesis.

## Figures and Tables

**Table 2:** Interaction partners of the HPV16 minor capsid protein L2 are listed in alphabetical order.

L2 Partner	Impact on HPV16 Entry	References
A2t	L2 interaction with S100A10 subunit of A2t promotes cellular uptake, trafficking to late endosomal multivesicular bodies (LE/MVBs) and protects the virus from lysosomal degradation.	[40,41,84]
Beta-actin	L2/actin interaction facilitates virus infection.	[139]
CCT complex	L2 interaction with the CCT3 subunit of the CCT chaperonin complex facilitates capsid disassembly.	[87]
Cep68	Interaction of the centrosomal protein 68 (Cep68) with L2 might facilitate virus infection.	[80]
Chromatin	L2 tethering to mitotic chromatin promotes nuclear entry. This interaction requires the L2 SUMO interaction motif within the central chromosome-binding region.	[73,126]
Cyclophilin B	Cyclophilin B contributes to initial conformational changes of L2 and dissociation of L1 and L2.	[22,94]
Dynein RanBP10	L2 interaction with subunits of the dynein motor complex, DYNLT1 and DYNLT3, in addition to L2 interaction with the DYNLT3/RanBP10/KPNA2 complex mediates minus-end-directed transport of the viral genome along microtubules towards and into the nucleus.	[123,124,127]
ESCRT	L2 interacts with ESCRT components Tsg101 and VPS4. ESCRT can form a complex with L1/L2 and is involved in endosome maturation to MVBs. TSG101 reduces the levels of L2 polyubiquitination.	[69,85]
Furin	Furin cleavage of L2 contributes to the priming of HPV. This can occur during virion morphogenesis, on the basement membrane or on the cell surface, contributing to the asynchronous uptake, interaction with γ-secretase, and subsequent trafficking.	[24,25,26,28,104,140]
Gamma-secretase	L2 binds to γ-secretase. A novel chaperone-like function of the γ-secretase enables membrane insertion of L2.	[104]
Hsc70	L2/Hsc70 chaperone interaction facilitates nuclear import. Hsc70 promotes the release of the vDNA/L2 from microtubules. Hsc/Hsp70 chaperones also interact with the L2/γ-secretase complex.	[104,109,123]
Membranes	L2´s membrane-destabilizing peptide or cell-penetrating peptide (CPP) near the C-terminus initiates membrane penetration until the transmembrane domain near the N-terminus of L2.	[100,101,102]
MYPOP	MYPOP senses incoming viruses via interaction with L2 and blocks viral early gene expression. L2/MYPOP interaction enhances MYPOP´s binding to the viral DNA (LCR).	[14]
Nuclear import receptor	L2 interacts with nuclear import receptors such as karyopherin alpha 2, enabling nuclear import not only during HPV16 morphogenesis but also during entry.	[127,128]
OBSL1	OBSL1 enables HPV endocytosis probably by linking the virus entry platform to the actin cytoskeleton. Further trafficking steps might be facilitated by L2/OBSL1 interaction.	[80]
p120-catenin	p120-catenin promotes L2/γ-secretase complex formation and L2 membrane insertion.	[103]
PML	L2´s SUMO interaction motif (L2 IVAL, aa 286–9) enables incorporation into newly formed PML NBs after mitosis.	[73,131]
Retromer	L2 interaction with retromer subunits VPS35 promotes endosome to Golgi trafficking and stabilizes membrane insertion of L2. L2/VPS35 enhances complex formation between retromer, Rab7, and TBC1D5, supporting disassembly of the retromer-HPV complex. L2 recruits retromer and retriever complexes via the same L2 domain.	[92,114,115,117,141]
Sorting Nexin 17 Sorting Nexin 27	L2/SNX17 interaction promotes trafficking to LE/MVBs, capsid disassembly, and protects L2 from lysosomal degradation. This interaction may support retromer recruitment. L2/SNX27 interaction facilitates trafficking probably by supporting retriever interaction.	[86,88,115,142]
SUMO	L2/SUMO covalent conjugation via the L2 SCM increases L2 stability and inhibits L1 binding. L2/SUMO interaction via the highly conserved SIM (L2 IVAL, aa 286–9) enhances L2 SUMOylation and PML NB association.	[131,132]
PATZ, PLINP, PMSP, TIN-Ag-RP	Functions of the interaction between HPV16 L2 protein and POZ-AT-Zn-finger protein (PATZ), papillomavirus L2 interacting nuclear protein (PLINP), papillomavirus minor structural protein interacting protein (PMSP), and tubular-nephritis antigen-related protein (TIN-Ag-RP) are unknown.	[143]
Tbx2 Tbx3	L2/Tbx interaction enhances repressive activity of T-box proteins Tbx2 and Tbx3 on the LCR as well as Tbx silencing of E6 expression.	[75]
TRAPPC8	L2/TRAPPC8 interaction may contribute to Golgi destabilization.	[83]

## Data Availability

Not applicable.
